# Elevated adipogenesis of marrow mesenchymal stem cells during early steroid-associated osteonecrosis development

**DOI:** 10.1186/1749-799X-2-15

**Published:** 2007-10-15

**Authors:** Hui H Sheng, Ge G Zhang, Wing Hoi WH Cheung, Chun Wai CW Chan, Yi Xiang YX Wang, Kwong Man KM Lee, Hong Fu HF Wang, Kwok Sui KS Leung, Ling L Qin

**Affiliations:** 1 Department of Orthopaedics & Traumatology, The Chinese University of Hong Kong, Hong Kong, China; 2 Department of Bone Metabolism, The Institute of Radiation Medicine, Fudan University, Shanghai, China; 3 Lee Hysan Clinical Research Laboratory, The Chinese University of Hong Kong, Hong Kong, China; 4 Department of Diagnostic Radiology & Organ Imaging, The Chinese University of Hong Kong, Hongkong, China

## Abstract

**Background:**

Increased bone marrow lipid deposition in steroid-associated osteonecrosis (ON) implies that abnormalities in fat metabolism play an important role in ON development. The increase in lipid deposition might be explained by elevated adipogenesis of marrow mesenchymal stem cells (MSCs). However, it remains unclear whether there is a close association between elevated adipogenesis and steroid-associated ON development.

**Objective:**

The present study was designed to test the hypothesis that there might be a close association between elevated adipogenesis and steroid-associated ON development.

**Methods:**

ON rabbit model was induced based on our established protocol. Dynamic-MRI was employed for local intra-osseous perfusion evaluation in bilateral femora. Two weeks after induction, bone marrow was harvested for evaluating the ability of adipogenic differentiation of marrow MSCs at both cellular and mRNA level involving adipogenesis-related gene peroxisome proliferator-activated receptor gamma2 (PPARγ2). The bilateral femora were dissected for examining marrow lipid deposition by quantifying fat cell number, fat cell size, lipid deposition area and ON lesions. For investigating association among adipogenesis, lipid deposition and perfusion function with regard to ON occurrence, the rabbits were divided into ON^+ ^(with at least one ON lesion) group and ON^- ^(without ON lesion) group. For investigating association among adipogenesis, lipid deposition and perfusion function with regard to ON extension, the ON^+ ^rabbits were further divided into sub-single-lesion group (SON group: with one ON lesion) and sub-multiple-lesion group (MON group: with more than one ON lesion).

**Results:**

Local intra-osseous perfusion index was found lower in either ON^+ ^or MON group when compared to either ON^- ^or SON group, whereas the marrow fat cells number and area were much larger in either ON^+ ^or MON group as compared with ON^- ^and SON group. The adipogenic differentiation ability of MSCs and PPARγ2 expression in either ON^+ ^or MON group were elevated significantly as compared with either ON^- ^or SON group.

**Conclusion:**

These findings support our hypothesis that there is a close association between elevated adipogenesis and steroid-associated osteonecrosis development.

## Background

Steroids are indicated for many inflammatory and autoimmune diseases, such as rheumatoid arthritis, systemic lupus erythematosus and severe acute respiratory syndrome. One of the most serious complications for steroid administration is osteonecrosis (ON), that most frequently presents in femoral heads and often advances to subchondral bone collapse and needs arthroplasty [1-3]. However, there is a high failure rate in steroid-associated ON patients [4]. Prevention of ON is a very important strategy. However, the unclear pathogenesis of ON is still the stumbling block for developing effective prevention modalities.

There are many postulations about the pathogeneses of steroid-associated ON. One of them is the theory of lipid deposition, i.e. the deposited marrow fat would compress on blood sinusoids to ischemia in compartmental bone: such as increased size of marrow fat cells, fat emboli and accumulation of lipid within the osteocytes [5,6]. However, the relationship between above observation and the increase in lipid deposition remains unexplained. One possibility is that marrow lipid was a consequence of the adipogenesis of marrow mesenchymal stem cells (MSCs) [7]. Results of a previous study showed increased number of small size fat cells in the early steroid-associated ON, that might be derived from the adipogenic differentiation of MSCs [8]. The in vitro studies also showed elevated adipogenic differentiation ability of MSCs after steroid treatment [9,10]. However, the relationship between the adipogenesis of marrow MSCs and steroid-associated ON remains unclear. The present study was designed specifically to compare the adipogenesis of MSCs between rabbits with ON and rabbits without ON, rabbits with single ON lesion and rabbits with multiple ON lesions using our established experimental model [11].

## Methods

### Animals and treatment

Twenty-five 28–30-week old male mature New Zealand White rabbits with body weight of 3.5–4.2 kg were used in this experiment. The ON induction procedure was done based on our established protocol [11]. Briefly, the rabbits were intravenously injected with 10 μg/kg body weight of lipopolysaccharide (LPS) (Escherichia coli 0111:B4, Sigma-Aldrich, Inc. USA). 24 hours later, three injections of 20 mg/kg body weight of methyprednisolone (MPS) (Pharmacia & Upjohn, USA) were given intramuscularly at a time interval of 24 hours. The rabbits were kept in cage and received a standard laboratory diet and had free access to food and water *ad libitum*. All animal experiment procedures described below were reviewed and approved by the animal ethics committee in the Chinese University of Hong Kong (Ref No.04/038/MIS).

### Dynamic-MRI for vessels perfusion function

Dynamic MRI for bilateral proximal femora and distal femora was done before LPS injection (week 0), one week (week 1) and two weeks (week 2) after MPS injection using a 1.5 T superconducting system (ACS-NT Intera; Philips, The Netherlands) based on our established protocol [11]. Briefly, rabbits were placed and fixed in supine position after anesthesia. Preliminary sagittal and oblique axial images were obtained to define the local longitudinal axis. The contrast-enhanced dynamic MR pulse sequence used previously established ultrafast T1-weighted gradient-echo sequences (turbo-field echo; Philips). A total of 200 dynamic images were obtained in 90s. A bolus of dimeglumin gadopentetate (Magnevist; Schering, Berlin, Germany) (0.8 mmol/kg/body weight) was rapidly administered automatically via the right ear vein, immediately followed by normal saline flush. Signal intensity (SI) was then measured in the regions of interest (ROIs) over the target site beneath the joint space in the mid-coronal T1-weighted images. The signal intensity values derived from the ROIs were plotted against time as time-intensity curve (TIC) using the Gyroview software system (Philips). The baseline value (SIbase) of the SI in a TIC was calculated as the mean SI value in the first three images. The maximum SI (SImax) was defined as the peak enhancement value at a given time interval of 90s after contrast injection. Perfusion parameter was calculated namely: "Maximum enhancement". "Maximum enhancement" was defined as the maximum percentage increase (SImax-SIbase) in SI from baseline (SIbase). The perfusion parameter was calculated according to the following equation:

Maximum enhancement=(SImax−SIbase)SIbase×100%
 MathType@MTEF@5@5@+=feaafiart1ev1aaatCvAUfKttLearuWrP9MDH5MBPbIqV92AaeXatLxBI9gBaebbnrfifHhDYfgasaacH8akY=wiFfYdH8Gipec8Eeeu0xXdbba9frFj0=OqFfea0dXdd9vqai=hGuQ8kuc9pgc9s8qqaq=dirpe0xb9q8qiLsFr0=vr0=vr0dc8meaabaqaciaacaGaaeqabaqabeGadaaakeaacqqGnbqtcqqGHbqycqqG4baEcqqGPbqAcqqGTbqBcqqG1bqDcqqGTbqBcqqGGaaicqqGLbqzcqqGUbGBcqqGObaAcqqGHbqycqqGUbGBcqqGJbWycqqGLbqzcqqGTbqBcqqGLbqzcqqGUbGBcqqG0baDcqGH9aqpdaWcaaqaaiabcIcaOiabbofatjabbMeajjabb2gaTjabbggaHjabbIha4jabgkHiTiabbofatjabbMeajjabbkgaIjabbggaHjabbohaZjabbwgaLjabcMcaPaqaaiabbofatjabbMeajjabbkgaIjabbggaHjabbohaZjabbwgaLbaacqGHxdaTcqaIXaqmcqaIWaamcqaIWaamcqGGLaqjaaa@64BA@

### MSCs adipogenesis evaluation

#### MSCs harvest and culture

After dynamic MRI measurement at week 2, the bone marrow was harvested from proximal femur for MSCs culture based on our established protocol [12]. MSCs were cultured in basal medium containing Dulbecco's modified Eagle's medium (DMEM) with 10% fetal bovine serum, 1% mixture of penicillin, streptomycin and neomycin (Invitrogen Corporation, Carlsbad, USA). The cells were cultured in an incubator at 37°C, 5% humidified CO_2 _for two weeks. Then the cells were harvested for the following evaluations:

#### MSCs adipogenesis evaluation

After plating cells to a 6-well plate (5000/cm^2^), the cells grew to 80% confluence. The adipogenic differentiation ability was induced in adipogenic medium for 10 days (15% normal horse serum and 100 nM dexamethasone in basal DMEM medium) [13]. First, the density of Oil Red O positive cells were calculated using Image Pro Plus software (Media Cybernetics Inc., Silver Spring, MD); Second, the intracellular lipid droplets were extracted and quantified. The cells were fixed with 10% neutral buffered formalin followed by incubating with 60% propylene glycol, then incubated with a newly filtered Oil Red O staining solution. After staining, the cells were rinsed with distilled water, and 1 ml of isopropyl alcohol was added to the stained dish. Aliquots of the extracted Oil Red O were measured at 510 nm with spectrophotometer (Ultrospec 3000, Pharmacia Biotech, USA) [14].

#### Adipogenic differentiation gene PPARγ2 expression

The cells after adipogenic induction were collected for PPARγ2 analysis. For RNA extraction, total RNA was isolated with TRIzol reagent (Gibco, USA). Single-stranded cDNA was then prepared from the total RNA extracted, using 100 units of M-MLV reverse transcriptase per reaction with an oligo-dT primer (Promega, Madison, USA). For PCR reaction, 1 ml of each cDNA was subjected to PCR reaction using rabbit PPARγ2 primers (PPARγ2 forward 5'CCAGGGGCCGAGAAGGAGA3' and reverse 5'AAGCCAGGGATGTTTTTG 3'). The internal control housekeeping gene GAPDH mRNA was also amplified under the same conditions to normalize PPARγ2 mRNA expression (GAPDH forward 5'GCGGAGCCAAAAGGGTCATCAT3' and reverse 5' CAGCCC CAGCATCGAAGGTAGAGG3'). PCR was performed in a DNA thermal circler (Biometra, Germany). The PCR products were electrophoresed on a 2% agarose gel in the presence of ethidium bromide and absorbance measured by densitometer (Bio-Rad, Model GS-670, USA). The ratio of PPARγ2 to GAPDH was calculated for quantitative comparison.

### Tissue preparation

The rabbits were euthanized with overdose pentobarbital sodium after bone marrow aspiration in two weeks. Bilateral femora were fixed for 3 days with 10% buffered formalin (Ph 7.4), then decalcified with 10% formic acid for 4 weeks. All the decalcified samples were embedded in paraffin, cut into 6 μm-thick sections along the coronal plane in the proximal one-third and axial plane for the distal part. Sections were stained with routine hematoxylin and eosin.

### Bone marrow fat cells measurement

Five sections from each animal were examined. Five fields (magnification 100×) within the proximal femur in each section were chosen. The first field was located at the approximate center of the femoral head at the ligamentum teres and the remaining four fields were located at the both sides of the first field. The mean of the five fields from each section was determined to represent that section. The mean of the five sections from each animal was taken as the value for that rabbit. The mean fat cells density, mean fat cells size and fat cells area would be measured with imaging process software Image-Pro Plus 5.1 (Media Cybernetics Inc., Silver Spring, MD). Fat cells density = marrow fat cells number in selected field/(selected field area – trabecular bone area); fat cells diameter = the total diameter of fat cells in selected field/the number of fat cells in selected field; fat cells area = the area of all fat cells in selected field/(the selected field area - trabecular bone area) [6,15].

### ON incidence and extension

The entire areas of each dissected part of bilateral femoral samples, including epiphysis and metaphysis, were examined for the presence of ON. Diagnosis of ON was blindly made by two pathologists based on the characteristic histopathological features with diffuse presence of empty lacunae or pyknotic nuclei of osteocytes in the bone trabeculae, accompanied by surrounding bone marrow necrosis [16]. All rabbits that had at least one ON lesion in the examined areas were considered to be ON^+^, while those without ON lesion were considered to be ON^-^. The ON^+ ^rabbits were further divided into sub-single-lesion group (SON group: with one ON lesion) and sub-multiple-lesion group (MON group: with more than one ON lesion).

### Statistics

The differences between ON^+ ^and ON^- ^group, MON and MON group were analyzed by nonparametric Mann-Whitney test using SPSS software 13.0 (SPSS Inc., Chicago, IL, USA). The results are expressed as the mean value ± standard of deviation. Statistical significance was set at *P *< 0.05.

## Results

### ON incidence and extension

**Figure 1:**
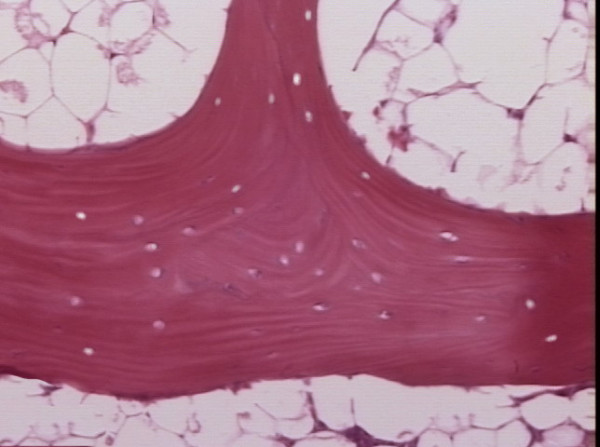
Histopathological features of osteonecrosis in ON+ group. The bone trabecular showed empty lacunae, surrounding by marrow tissue with necrotic marrow cell debris(Hematoxylin & Eosin, 200×).

No rabbits died during the entire experiment period. Of the 25 rabbits, 15 were found ON^+ ^(60%) and 10 were ON^- ^(40%). Of the 15 ON^+ ^rabbits, 6 rabbits had only one ON lesion and were classified to SON group, 9 rabbits had more than one lesion and were classified into MON group. Histologically, ON lesion showed accumulation of marrow fat cells debris and bone trabeculae with many empty lacunae (Figure 1).

### Dynamic MRI for perfusion function

**Figure 2:**
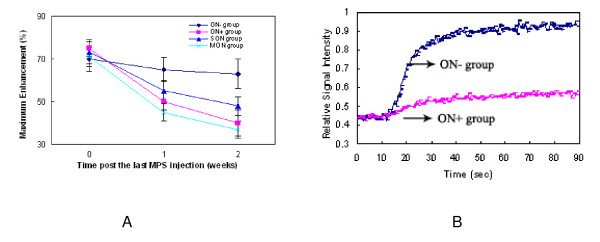
Blood perfusion assessed by dynamic MRI for Maximum Enhancement and Time-Signal Intensity. (A) Maximum Enhancement at the examined sites (both proximal femora and distal femora, the similar pattern was found, data not shown here for distal femora) showed a significant decrease from baseline in ON^+ ^rabbits at week 2 after steroid induction. There were significant decrease in Maximum Enhancement between ON^+ ^and ON^-^group, MON and SON group at week 2 (p < 0.05). (B) Representative Time-Signal Intensity curves from contrast-enhanced dynamic MRI on proximal femur. The Time-Signal Intensity curve showed a significant decrease in enhancement slope in ON^+ ^group as compared with ON^- ^group at week 2.

For "Maximum Enhancement" in proximal femora, the rabbits in ON^+ ^and MON group showed a continuous decrease with time. The ON^+ ^group showed a 36.5% decrease as compared with ON^- ^rabbits (p < 0.05). The MON group showed a 20% decrease as compared with SON group (p < 0.05) at week two (Figure 2); Similar pattern was found in distal femora (data not shown here).

### Bone marrow lipid deposition

#### Fat cells density

The fat cells density was 265 ± 23/mm^2 ^in ON^+ ^group, increased by 47.2% as compared with ON^- ^group (180 ± 19/mm^2^) (p < 0.05). It was 289 ± 28/mm^2 ^in MON group, much larger as compared with SON group (240 ± 26 mm^2^) (p < 0.05).

#### Fat cells size

The mean fat cells diameter was 40.3 ± 4.1 mm in ON^+ ^group, and 45.8 ± 5.3 mm in ON^- ^group (p > 0.05). There were no significant difference found in fat cells size between SON and MON group (p > 0.05).

#### Fat cells area

**Figure 3:**
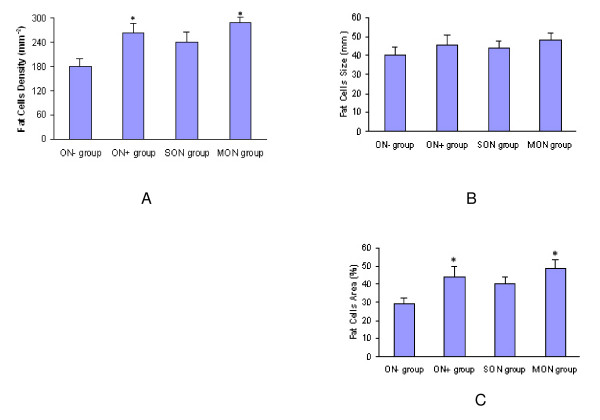
Bone marrow fat deposition feature in different groups. (A) Significant increase in fat cells density in ON^+ ^and MON group as compared with ON^- ^and SON group (P < 0.05); (B) There were no significant change in fat cells size between ON^+ ^and ON^- ^group, MON and SON group (p > 0.05); (C) Significant increase in fat cells area in ON^+ ^and MON group as compared with ON^- ^and SON group respectively (p < 0.05).

The fat cells area was 43.7 ± 5.7% in ON^+ ^group, which was 49.7% larger than ON^- ^group (29.2 ± 3.2%) (P < 0.05); The fat cells area in MON group was 48.6 ± 5.1%, which was 20% larger than SON group (40.2 ± 3.7%) (P < 0.05) (Figure 3).

### Adipogenic differentiation ability and PPARγ2 gene expression

#### Adipogenic differentiation ability

**Figure 4:**
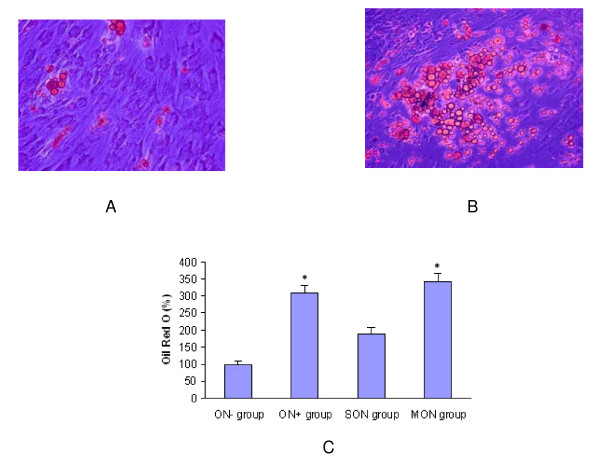
Adipogenesis of MSCs from different groups. Representative pictures showing much more adipocyte-like cells formation in ON^+ ^group (B) than ON^- ^group (A)(Oil Red O staining 100×); (C) Quantification result showed much more lipid droplets in ON^+ ^and MON group as compared with ON^- ^and SON group (p < 0.05).

The cells accumulated triglycerides vesicles, that was small initially and increased in size with time. The number of adipocytes in ON^+ ^group was 270% more as compared with ON^- ^group, 120% more in MON group as compared with SON group(p < 0.05). The optical density results showed 210% more triglycerides formation in ON^+ ^group as compared with ON^- ^group, and 80% more in MON group as compared with SON group (p < 0.05) (Figure 4).

#### PPARγ2 gene expression

The PPARγ2 mRNA expression in ON^+ ^group was 180% higher as compared with ON^- ^group (p < 0.05), and 85% higher in MON group as compared with SON group (p < 0.05) (Table 1).

**Table 1:** PPAR γ 2 mRNA expression in MSCs of different groups.

Groups	PPAR γ
ON^+ ^group	49.0 ± 2.41*
ON^- ^group	17.5 ± 1.90
MON group	61.7 ± 1.75^#^
SON group	34.3 ± 2.30

Note: Values are expressed as the ratio of PPAR γ to internal control GAPDH (mean ± SD).

* *p *< 0.05, compared with ON^-^group; # p < 0.05, compared with SON group.

## Discussion

The present study provides for the first time the evidence on a close association between the adipogenesis of MSCs and steroid-associated ON development during early stage.

A close association between elevated adipogenesis of MSCs and steroid-associated osteonecrosis occurrence. In the present study, the MSCs showed elevated adipogenenic differentiation ability at cellular and molecular level in ON^+ ^group as compared with ON^- ^group. The histological evidence showed increased lipid deposition including larger fat cells number and fat deposition area in ON^+ ^group as compared with ON^- ^group. These suggested that the accumulation of marrow fatty tissue might come from the differentiation of MSCs [6]. At the same time, the local blood perfusion function in ON^+ ^group was significant diminished at a time-dependent pattern. Bone marrow lipid deposition would affect blood perfusion function even to ischemia [17,18]. These evidences showed the elevated adipogenesis of MSCs was associated with steroid-associated ON occurrence.

A close association between elevated adipogenesis of MSCs and steroid-associated osteonecrosis extension. In this study, the ON^+ ^rabbits were further divided into SON and MON group based on the ON extension. The marrow MSCs showed higher adipogenic differentiation ability in MON group as compared with SON group. The histological evidence showed increased lipid deposition including larger fat cells number and fat deposition area in MON group as compared with SON group. These showed that the ability of adipogenic differentiaon of MSCs increased with larger ON extension. At the same time, the intraosseous blood perfusion in MON group was significant decreased at a time-dependent pattern as compared with SON group. These evidences showed the elevated adipogenesis of MSCs was associated with steroid-associated ON extension.

There were few published works exploring the relationship between adipogenesis of MSCs and steroid-associated ON. Lee studied the adipogenic ability of MSCs from ON patients was not able to find significant change. This difference between Lee and our present study may be explained by the two reasons: First, the samples in the patients study were in a much advanced stage as compared with the ON rabbit model histopatholocially, for they were receiving hip replacement surgery; Second, the adipogenesis ability of MSCs in osteoarthritis(OA) patients might have been elevated, this might blunt the difference between OA and ON patients [19,20]. The adipogenesis of MSCs, including the colony-forming unit of adipocytes was not compared between before and after steroid administration in this study. As clinical study showed core decompression would relieve ON development, marrow aspiration before steroid administration might affect ON development in the rabbit model. This is one of the limitations of this study. This study showed that there is a close association between elevated adipogenesis of MSCs and steroid-associated ON development.
